# Defining the spatial distribution of extracellular adenosine revealed a myeloid-dependent immunosuppressive microenvironment in pancreatic ductal adenocarcinoma

**DOI:** 10.1136/jitc-2022-006457

**Published:** 2023-08-08

**Authors:** Vincenzo Graziano, Andreas Dannhorn, Heather Hulme, Kate Williamson, Hannah Buckley, Saadia A Karim, Matthew Wilson, Sheng Y Lee, Brajesh P Kaistha, Sabita Islam, James E D Thaventhiran, Frances M Richards, Richard Goodwin, Rebecca Brais, Jennifer P Morton, Simon J Dovedi, Alwin G Schuller, Jim Eyles, Duncan I Jodrell

**Affiliations:** 1 Cancer Research UK Cambridge Institute, University of Cambridge, Cambridge, UK; 2 Cancer Research UK Cambridge Centre, University of Cambridge, Cambridge, UK; 3 Imaging and Data Analytics, Clinical Pharmacology and Safety Sciences (CPSS), AstraZeneca R&D, Cambridge, UK; 4 Medical Research Council Toxicology Unit, University of Cambridge, Cambridge, UK; 5 Cancer Research UK Beatson Institute, Glasgow, UK; 6 Oncology R&D, Research and Early Development, AstraZeneca R&D, Cambridge, UK; 7 Department of Oncology, University of Cambridge, Cambridge, UK; 8 Department of Pathology, Cambridge University Hospitals NHS Foundation Trust, Cambridge, UK; 9 School of Cancer Sciences, University of Glasgow, Glasgow, UK; 10 Oncology, AstraZeneca R&D Boston, Waltham, Massachusetts, USA

**Keywords:** Adenosine, Macrophages, Tumor Microenvironment, Immunity, Innate, Immunotherapy

## Abstract

**Background:**

The prognosis for patients with pancreatic ductal adenocarcinoma (PDAC) remains extremely poor. It has been suggested that the adenosine pathway contributes to the ability of PDAC to evade the immune system and hence, its resistance to immuno-oncology therapies (IOT), by generating extracellular adenosine (eAdo).

**Methods:**

Using genetically engineered allograft models of PDAC in syngeneic mice with defined and different immune infiltration and response to IOT and autochthonous tumors in KPC mice we investigated the impact of the adenosine pathway on the PDAC tumor microenvironment (TME). Flow cytometry and imaging mass cytometry (IMC) were used to characterize the subpopulation frequency and spatial distribution of tumor-infiltrating immune cells. Mass spectrometry imaging (MSI) was used to visualize adenosine compartmentalization in the PDAC tumors. RNA sequencing was used to evaluate the influence of the adenosine pathway on the shaping of the immune milieu and correlate our findings to published data sets in human PDAC.

**Results:**

We demonstrated high expression of adenosine pathway components in tumor-infiltrating immune cells (particularly myeloid populations) in the murine models. MSI demonstrated that extracellular adenosine distribution is heterogeneous in tumors, with high concentrations in peri-necrotic, hypoxic regions, associated with rich myeloid infiltration, demonstrated using IMC. Protumorigenic M2 macrophages express high levels of the Adora2a receptor; particularly in the IOT resistant model. Blocking the in vivo formation and function of eAdo (Adoi), using a combination of anti-CD73 antibody and an Adora2a inhibitor slowed tumor growth and reduced metastatic burden. Additionally, blocking the adenosine pathway improved the efficacy of combinations of cytotoxic agents or immunotherapy. Adoi remodeled the TME, by reducing the infiltration of M2 macrophages and regulatory T cells. RNA sequencing analysis showed that genes related to immune modulation, hypoxia and tumor stroma were downregulated following Adoi and a specific adenosine signature derived from this is associated with a poorer prognosis in patients with PDAC.

**Conclusions:**

The formation of eAdo promotes the development of the immunosuppressive TME in PDAC, contributing to its resistance to conventional and novel therapies. Therefore, inhibition of the adenosine pathway may represent a strategy to modulate the PDAC immune milieu and improve therapy response in patients with PDAC.

**WHAT IS ALREADY KNOWN ON THIS TOPIC**

- The adenosine pathway generates extracellular adenosine and its components are known to be overexpressed in tumors. Extracellular adenosine is recognized as an immune suppressive molecule. CD73, the member of the pathway that enables the final step of conversion of AMP to adenosine, is highly expressed on the cancer cell surface in many tumors, including pancreatic ductal adenocarcinoma (PDAC).

**WHAT THIS STUDY ADDS**

- Here, we show that in murine models of PDAC, the adenosine pathway is overexpressed by a population of myeloid immune cells. We visualized adenosine spatially in the tumor microenvironment of a relevant, preclinical model of PDAC, identifying that its distribution is mostly confined to myeloid-rich, hypoxic areas. We discovered that protumorigenic myeloid populations (in particular, M2 macrophages) represent the target of adenosine stimulation (Adora2a expressing) and therefore it is responsible for the formation and maintenance of an immune suppressive microenvironment. We were able to generate a specific, transcriptomic signature from our preclinical experiments that predicts survival in patients with PDAC. Finally, we demonstrated that inhibiting the adenosine pathway improved response to cytotoxic and immunotherapy drugs in murine PDAC models.

**HOW THIS STUDY MIGHT AFFECT RESEARCH, PRACTICE OR POLICY**

- This study unveils a previously unknown myeloid-dependent axis of immunosuppression in PDAC and could inform future clinical trials that will evaluate inhibitors of the adenosine pathway. Such studies might improve outcomes for patients with PDAC, a major unmet clinical need.

## Introduction

Survival for patients with pancreatic ductal adenocarcinoma (PDAC) has not changed significantly in the last 50 years and remains poor (https://www.cancerresearchuk.org/health-professional/cancer-statistics-for-the-uk).^1^ There is a need for new treatments, given that the current standard of care for patients with metastatic disease is associated with poor outcomes, with less than 10% of patients living for more than 2 years.^2^ In addition to relative resistance to conventional therapies, cancer immunotherapy (immuno-oncology therapy, IOT) is also ineffective in this disease, except in a small group of patients (1–2%) with microsatellite instability/mismatch repair deficient (MSI-H/dMMR) tumors.^3^ Several authors consider that the reason for this resistance can be ascribed to the low mutational burden of this neoplasm, which leads to lymphocyte exclusion and anergy.^4 5^ However, the tumor microenvironment (TME) in PDAC has also been shown to be populated by a rich variety of immune cells, most of which demonstrate immune suppressive features, which contribute to the resistance to immunotherapy.^6^

The adenosine pathway is an immunosuppressive axis which has gained much attention in cancer immunology for its role in suppressing the immune activation associated with cytotoxic treatments (chemotherapy, targeted therapy and radiotherapy).^7–9^ This has led to the clinical evaluation of inhibitors of the pathway in combination with more conventional approaches.^10^ The adenosine pathway involves conversion of extracellular ATP, a powerful immune activator, to extracellular adenosine (eAdo) by the ectonucleotidases CD39 and CD73.^11^ eAdo has been linked to cancer in several studies that have demonstrated that its concentration in different tumor tissues is several folds higher than in normal tissues.^7 12^ CD39 is overexpressed in a subpopulation of exhausted tumor-infiltrating T cells^13 14^ and its expression correlates with another marker of immunosuppression (Programmed cell death protein 1 or PD-1 expression).^14^ CD39 and CD73 have roles in the aggressiveness of adult glioblastoma,^15^ where they are expressed on infiltrating macrophages.^16 17^ The adenosine signature recently published by Sidders and colleagues^18^ shows that this pathway correlates with resistance to immunotherapies and is associated with other genetic features of tumor aggressiveness, such as p53 mutations. The abundant presence of eAdo in the microenvironment can dampen immune activation through the stimulation of a protumorigenic stroma. This is mostly orchestrated by macrophages and myeloid derived suppressive cells (MDSCs),^19 20^ favoring a tolerogenic function of dendritic cells (DCs)^21–23^ which results in inhibition of T cells/natural killer (NK) cell activation.^24 25^

The myeloid populations play a pivotal role in the aggressiveness of many cancer types and in particular, PDAC. For instance, the presence of protumorigenic populations of macrophages^26 27^ and MDSCs^28^ infiltrating the microenvironment, is associated with poor survival and correlates with immune exclusion of PDAC. Macrophages can elicit the secretion of cytokines which can on the one hand, favor the proliferation and invasiveness of cancer cells while interacting with cancer-associated fibroblasts,^29–31^ and on the other hand induce anergy and physical exclusion of adaptive immune cells.^32–34^ Targeting macrophages in a preclinical pancreatic cancer model has been demonstrated to be effective to obtain tumor regression and reduce metastatic formation.^35^ Unfortunately, this approach has not translated into clinical benefit, which in part can be explained by the fact that the global reduction of the tumor-infiltrating macrophages can be biologically different from reprogramming distinct tumor associated macrophage subtypes.^31^

Some recent publications link the adenosine pathway to the biology and aggressiveness of PDAC. PDAC has been shown to have an increased adenosine pathway RNA signature associated with a worse prognosis,^18^ and genes encoding for the receptors for eAdo as well as CD73 have been found to be overexpressed in bulk-RNA sequencing (RNAseq) when comparing tumors to normal pancreatic tissue.^8^ A role for eAdo in shaping myeloid response to PDAC has recently been suggested. Using genetic manipulation of CD73 in cancer cells and mice, Jacoberger-Foissac and colleagues demonstrated that CD73 can be overexpressed in a percentage of tumor-infiltrating myeloid cells other than cancer cells, contributing to infiltration of M2 macrophages in KPC mice.^36^ King and colleagues highlighted that genetic alteration of CD73 impaired the secretion of GM-CSF (Granulocyte-macrophage colony-stimulating factor), reducing myeloid-derived suppressive cell infiltration in PDAC mouse models.^37^ Also, it has been suggested that genetic alteration of CD73 in cancer cells induces a modest sensitivity to gemcitabine treatment in vitro. However, little is known about the complex mechanism generated by the adenosine pathway resulting in the immunosuppressive characteristics of pancreatic cancer microenvironment and stroma, in particular the role that the adenosine pathway and its therapeutic inhibition have in shaping the immune infiltration of this disease.

Here, we propose a model where the tumor-infiltrating immune cell populations of PDAC generate an axis of immunosuppression, where eAdo produced mostly in hypoxic regions of the tumor (identified using mass spectrometry imaging, MSI), enriched for the myeloid cell infiltration, stimulates protumorigenic M2 macrophages. The axis described is expressed preferentially by the IOT-resistant model when compared with the IOT-responsive one. Therefore, blocking the adenosine pathway in the IOT-resistant PDAC model, strongly suppresses the formation of eAdo and reshapes the immune microenvironment, favoring disease control when combined with cytotoxic treatments and immunotherapies. Bulk RNAseq gene analysis confirms the role of the myeloid-dependent adenosine pathway in PDAC survival, underpinning the importance of our results for understanding the biological complexity and the clinical utility of the adenosine pathway inhibition.

## Methods and materials

### Cell lines and chemicals

*Kras*^LSL-G12D/+^; *Trp53*^LSL-R172H/+^; *Pdx1*-Cre; *Rosa26*^YFP/YFP^ (KPCY)-derived cell lines 2838c3, 6499c4, 6620c1 (IOT-responsive), 6419c5, 6694c2 and 6422c1 (IOT-resistant) were a kind gift from Ben Stanger (University of Pennsylvania). The cell lines were obtained from single cell cloning strategy, as described previously, and were generated from tumors developed in KPCY mice on a C57BL/6 background.^38^ PANC-1 was used for in vitro experiments as a representative human PDAC cell line. Cells were grown up to 20 passages in DMEM (Dulbecco's modified eagle medium medium with pyruvate, L-glutamine and D-glucose; Gibco, #41966029) supplemented with 5% fetal bovine serum (FBS) (Gibco, #10270106). All the cell lines were analyzed for short tandem repeat (STR) fingerprinting and tested for Mycoplasma routinely.

### Mice and in vivo experiments

Tumor allograft experiments were performed in the animal facility (Biological Resource Unit, BRU) of the CRUK Cambridge Institute, in accordance with the UK Animals Scientific Procedures Act 1986, with approval from the CRUK Cambridge Institute Animal Ethical Review and Welfare Body (ref. n. PP6047951 and PPL7008363). 8–12 weeks old female C57BL/6 mice were used for in vivo experiments and were purchased from Charles River (UK).

Tumor allograft studies were performed with technical assistance from CRUK-CI BRU staff. Mice were subcutaneously injected in the right flank with 1×10^6^ KPCY-derived cells in 50% phosphate-buffered saline (PBS) and 50% Matrigel basement membrane matrix (#354234, Corning). In the interventional experiments, mice were treated as indicated, starting 12–14 days from tumor cell implantation, to allow the microenvironment to establish. Tumor volume was calculated using the formula; (π/6)×(width)^2^×length. Tumor response was defined based on the % of change of the longest diameter from start of therapy (stable disease (SD) <20% increase and <30% decrease of target lesion according to the response evaluation criteria in solid tumours or RECIST v. 1.1). Mice were then killed at specific endpoints (eg, 14 days from start of treatment) or when the tumor reached 2000 mm^3^ (or before in case of appearance of clinical signs).

*Kras*^LSL-G12D/+^; *Trp53*^LSL-R172H/+^; *Pdx1*-Cre (KPC) mice for tumor phenotyping, were obtained from a breeding colony maintained by the CRUK-CI Genome Editing Core team. Tumors were detected by palpation followed by ultrasound imaging by the Genome Editing Core. Tumor tissues, spleens and mesenteric and inguinal lymph nodes from KPC mice were provided once tumor dimensions or health status rendered them unsuitable for therapeutic studies. KPC mice were killed when showing clinical signs of the disease (swollen abdomen, loss of body conditioning resembling cachexia, reduced mobility).

KPC mice used for the interventional study were bred at the CRUK Beatson Institute and maintained on a mixed background. All work was performed under UK Home Office license and approved by the University of Glasgow Animal Welfare and Ethical Review Board (ref. n. PP8411096). Mice of both sexes, in similar proportions, were used in all cohorts. Mice suspected to have PDAC following palpation were anesthetized in 0.2 L/min medical air and isoflurane and then underwent three-dimensional (3D) ultrasound imaging using the VisualSonics Vevo 3100 ultrasound system with MX550D 40 µm resolution transducer (Fujifilm). Mice were randomly assigned to one of the four treatment groups (A: vehicles+isotype; B: AZD6738+gemcitabine; C: AZD4635+2c5mIgG1; D: AZD6738+gemcitabine + AZD4635+2c5mIgG1) once tumors were confirmed by imaging, and follow-up scans were performed weekly until endpoint was reached. Schedule of the treatments are specified in the results and figures sections. Mice were culled when exhibiting moderate symptoms of pancreatic cancer (see above). Statistical assessment of survival was carried out by Kaplan-Meier and log-rank analysis. Analysis of ultrasound images was performed using Vevo Lab software (V.3.1.1) from VisualSonics. In 3D mode, stacked images of the tumor were imported and the tumor border annotated, allowing a 3D construct to be formed.

When indicated the following drugs were used: AZD6738 (ATRi; 25 mg/kg daily for 4 days), AZD4635 (Adora2ai; 50 mg/kg two times a day), 2c5mIgG1 (anti-CD73; 10 mg/kg two times a week), AB740080 D265A (anti-PD-L1: 10 mg/kg two times a week), NIP228 mouse IgG1 control kappa (isotype; 10 mg/kg two times a week) and NIP228 muIgG1 D265A (isotype; 10 mg/kg two times a week) were provided by AstraZeneca. The antibody anti-CD73 2c5mIgG1 is a murine IgG1 with minimal Fc mediated activity^39^; gemcitabine hydrochloride (Tocris, 3259) was used at 100 mg/kg two times a week; InVivoPlus anti-CD40 (clone FGK4.5/FGK45; Bio X Cell BE0016-2) and InVivoPlus rat IgG2a isotype control, anti-trinitrophenol (clone 2A3; Bio X Cell BE0089) were used as a single injection of 100 µg. InVivoPlus anti-CTLA-4 (clone 9H10; Bio X Cell BP0131) or InVivoPlus isotype control polyclonal Syrian hamster IgG (Bio X Cell BP0087): 200 µg/dose×three times.

### Clonogenic assay

KPCY-derived cells were seeded at 200 cells/well in a 6-well plate. After 24 hours, cells were treated with different concentrations of anti-CD73 (2c5mIgG1) antibody (1, 10, 100 µg/mL) or isotype (NIP228 mouse IgG1 control kappa) for 8 days. Antibodies were added every 3 days. On day 8, colonies were stained following the sulforhodamine (SRB) protocol previously described.^40^ Images were taken and analyzed using GelCount (Oxford Optronix). Colony forming efficiency was calculated as a ratio between the number of colonies and number of plated cells. Surviving fractions were calculated as the ratio between wells treated with anti-CD73 antibody and the ones treated with isotype. At least three wells per condition were plated for each of the three replicates per experiment.

### Incucyte time lapse imaging

KPCY-derived cells were plated at 2500 cells/well density in a 96-well black-wall plate (at least three wells per condition). Cells were grown in cell culture medium supplemented with the indicated concentration of anti-CD73 (2c5mIgG1) or isotype (NIP228 mouse IgG1 control kappa) antibody in triplicate. Images were acquired with 10× objective, every 3 hours from three different fields per well using Incucyte Live cells imaging microscope (Essen BioScience). Confluence was calculated as the average of the three fields using the Incucyte algorithm. Experiments were repeated at least three times.

### Human cell line viability assay

For viability experiments, 3000 PANC-1 cells/well were plated into a 96-well plate overnight and then dosed with IC50 for each compound (gemcitabine 0.5 µM; oxaliplatin 30 µM; docetaxel 0.5 nM; 5-FU 52 µM; cisplatin 20 µM) either alone or in combination with oleclumab at 1 nM. For combination and pretreatment experiments, cells were either pretreated with oleclumab at 1 nM for indicated time (2 hours or 24 hours) before addition of chemotherapies at IC50s. Viability was assessed using CellTiter-Glo as per manufacturer’s instructions at either 3 or 7 days and expressed as percentage of cells in the treated wells over control. Oleclumab (MEDI9447) was kindly provided by AstraZeneca.

### Single cell suspension preparation

For experiments in subcutaneous (s.c.) allografts, tumors were weighed and placed in RPMI and finely minced with scissors in a 2 mL tube, which was then washed with up to 2.5 mL of digestion buffer (Tumor Dissociation Kit, Miltenyi, 130-096-730) plus deoxyribonuclease I (300 µg/mL, Sigma, DN25-1G). Dissociation was performed using the protocol suggested by Miltenyi. For KPC tumors, a trypsin inhibitor (250 µg/mL, Sigma, T6522) was added to the digestion buffer. Following the digestion, the samples were passed through a 70 µM strainer filter (Greiner Bio-One, 542–070) washed with MACS buffer (PBS+0.1% FBS and 2 nM EDTA).

Mouse spleens, inguinal and mesenteric lymph nodes were mashed on a 100 µM filter (Greiner Bio-One, 542–000) over a 50 mL tube, using a syringe plug and the filter was washed with MACS buffer and centrifuged (at 4°C as for all the following centrifugation). Red cell lysis buffer (1 mL; 0.15M ammonium chloride; 10 mM potassium hydrogen carbonate; 0.1 mM EDTA; pH 7.4 (adjusted with KOH) was then used to resuspend splenocytes, 3 min at room temperature and then washed with MACS buffer and centrifuged at 300 g for 5 min. Samples were eventually resuspended in 200–400 µL of MACS buffer.

### Flow cytometry

Single cell suspensions were aliquoted in a round-bottom 96-well/plate (CoStar, 3879) and stained with live/dead fixable stain (Invitrogen, L34962; 1:100 in PBS) for 10 min at room temperature. After washing in MACS buffer, cells were FC-blocked with anti-CD16/32 antibody (BioLegend Cat# 101320, RRID:AB_1574975; 1:100) for 5 min. Then, antibodies for surface staining were added and incubated at 4°C for 30 min. After washing, cells were fixed with FACS (fluorescence-activated cell sorting) fix buffer (PBS+1% formaldehyde+0.02 g/mL glucose+0.02% sodium azide) for 10 min and then washed and resuspended in MACS buffer for FACS analysis. For intracellular staining, cells were fixed with fixation/permeabilization buffer (Invitrogen 00-5123-43 and 00-5223-56) for 15 min, then washed in perm buffer and stained with the relevant antibody in perm buffer for 60 min. Cells were then washed and resuspended in MACS buffer for FACS analysis. Samples were acquired using BD-Symphony flow cytometer and the generated FCS files were analyzed using FlowJo V.10 software (RRID:SCR_008520). The following antibodies were used and gating strategies are shown in the Online supplemental figures (myeloid populations and lymphoid population; online supplemental figure 1A,B respectively): BV786-CD45 (BD Biosciences Cat# 564225, RRID:AB_2716861; 1:200), APC/Fire750-CD3 (BioLegend Cat# 100248, RRID:AB_2572118; 1:50), BV650-CD8 (BioLegend Cat# 100741, RRID:AB_11124344; 1:100), BV711-CD4 (BioLegend Cat# 100549, RRID:AB_11219396; 1:200), APC-Foxp3 (Invitrogen Cat# 17-5773-82, RRID:AB_469457; 1:100), FITC-CD19 (BioLegend Cat# 115505, RRID:AB_313640; 1:200), BV510-CD11b (BioLegend Cat# 101245, RRID:AB_2561390; 1:200), PerCP/Cy5.5-CD44 (BioLegend Cat# 103031, RRID:AB_2076206; 1:200), BV421-PD-1 (BioLegend Cat# 135218, RRID:AB_2561447; 1:100), BV421-PD-L1 (BioLegend Cat# 124315, RRID:AB_10897097; 1:100), PE/Cy7-CD39 (BioLegend Cat# 143805, RRID:AB_2563393; 1:100), PE-CD73 (BioLegend Cat# 127206, RRID:AB_2154094; 1:100), FITC-F4/80 (BioLegend Cat# 123108, RRID:AB_893502; 1:200), PE/Cy7-CD206 (BioLegend Cat# 141719, RRID:AB_2562247; 1:100), PerCP/Cy5.5-Ly6C (BioLegend Cat# 128012, RRID:AB_1659241; 1:100), APC/Cy7-Ly6G (BioLegend Cat# 127624, RRID:AB_10640819; 1:100), AF700-MHC-II (BioLegend Cat# 107629, RRID:AB_2290801; 1:200), PE-CD11c (eBioscience Cat# 12-0114-83, RRID:AB_465553; 1:200), BV605-NKp46 (BioLegend Cat# 137619, RRID:AB_2562452; 1:25), APC-Adora2a (Novus Biotech Cat# NBP1-39474APC; 1:150), APC-CD86 (BioLegend Cat# 105012, RRID:AB_493342; 1:100). Gating strategy for immune subpopulations is shown in online supplemental figure 1A,B. For CD73 in vitro staining of KPCY-derived cell lines, cells were stained as described above with live/dead fixable stain and PE-CD73 antibody and analyzed with BD-Symphony flow cytometer. For in vitro treatment, cells were treated with anti-CD73 (2c5mIgG1) or isotype (NIP228) antibody at a concentration of 10 µg/mL for 24 hours. Competitive staining was performed before this experiment to confirm there was no competition between 2c5mIgG1 and TY11.8 (PE-CD73) clones (data not shown).

10.1136/jitc-2022-006457.supp1 Supplementary data

### Tissue preparation for mass spectrometry imaging and imaging mass cytometry analysis

PDAC mouse tumors were snap frozen in liquid nitrogen immediately after resection and the tissues were embedded in a hydroxypropyl methylcellulose (HPMC)/polyvinylpyrrolidone (PVP) hydrogel as previously described.^41^ Sectioning was performed on a CM3050 S cryostat (Leica Biosystems, Nussloch, Germany) at a section thickness of 10 µm and the tissue sections were immediately thaw mounted and dried under a stream of nitrogen and sealed in vacuum pouches to preserve the metabolic integrity of the sections. Tissue sections for DESI (Desorption Electrospray Ionization)-MSI and imaging mass cytometry (IMC) were thaw-mounted onto Superfrost microscope slides (Thermo Scientific Waltham, Massachusetts, USA), while sections prepared for MALDI (Matrix-Assisted Laser Desorption Ionization)-MSI were thaw mounted onto conductive ITO coated slides (Bruker Daltonik, Bremen, Germany). PVP (MW 360 kDa) and HPMC (viscosity 40–60 cP, 2% in H2O (20 C) were purchased from Merck (Darmstadt, Germany). Methanol, water, isopentane and isopropyl alcohol were obtained from Fisher Scientific (Waltham, Massachusetts, USA).

### Mass spectrometry imaging

DESI-MSI analysis was performed on a Q-Exactive mass spectrometer (Thermo Scientific, Bremen, Germany) equipped with an automated two-dimensional-DESI ion source (Prosolia, Indianapolis, Indy, USA) operated in negative ion mode, covering the applicable mass range up to an m/z of 1000, with a nominal mass resolution of 70,000. The injection time was fixed to 150 ms resulting in a scan rate of 3.8 pixel/s. The spatial resolution was adapted between experiments to allow acquisition of the data for all directly compared samples within a single experiment of 48 hours, with pixel sizes ranging from 35 to 75 µm. A home-built Swagelok DESI sprayer was operated with a mixture of 95% methanol, 5% water delivered with a flow rate of 1.5 µL/min and nebulized with nitrogen at a backpressure of 6 bar. The resulting .raw files were converted into .mzML files using ProteoWizard msConvert^42^ (V.3.0.4043) and subsequently compiled to an .imzML file (imzML converter^43^ V.1.3). All subsequent data processing was performed in SCiLS Lab (V.2021b, Bruker Daltonik, Bremen, Germany).

MALDI-MSI analysis was performed on a rapifleX Tissuetyper instrument (Bruker Daltonik, Bremen, Germany) operated in negative detection mode. 9-Aminoacridine prepared in 80:20 methanol:water was used as an MALDI matrix and spray deposited using an automated spray system (M3-Sprayer, HTX technologies, Chapel Hill, North Carolina, USA). MALDI experiments were performed with a spatial resolution of 50 µm. A total of 400 laser shots were summed up per pixel to give the final spectra. For all experiments the laser was operated with a repetition rate of 10 kHz. All raw data were directly uploaded and processed in SCiLS lab (V.2021b) software packages. All DESI and MALDI data and images were normalized to the total ion current to compensate for signal variation across the course of the experiments. Data segmentation pipeline is shown in online supplemental materials and methods.

10.1136/jitc-2022-006457.supp2 Supplementary data

### Imaging mass cytometry

IMC was performed on a slide which had already been analyzed by DESI-MSI. Antibodies used for IMC staining are shown in online supplemental table 1. Untagged antibodies were tagged in house, using Fluidigm Maxpar Antibody Labeling Kit, according to manufacturer’s instructions. Following DESI-MSI analysis, the slide was fixed with 4% paraformaldehyde in PBS for 10 min. The slide was washed 3×5 min in PBS, permeabilized for 5 min with 1:1000 dilution of Triton X-100 in casein solution, washed 3×5 min in PBS, and blocked for 30 min with casein solution. Antibodies were diluted to an appropriate concentration in casein solution and the slide incubated overnight with the antibody solution at 4°C. The slide was washed 3×5 min in PBS and nuclei were stained with DNA intercalator-iridium at a dilution of 1:400 in PBS for 30 min. The slide was washed 3×5 min in PBS, 30 s in deionized water, then dried for storage at room temperature until analysis. A region for IMC analysis was selected using consecutive H&E stained sections and the DESI-MSI results. A box with approximately 2×1.8 mm area was selected for analysis to include necrotic, necrotic margin and viable tumor regions. IMC analysis was performed using a Hyperion instrument (Fluidigm Corporation, San Francisco, California, USA) with an ablation energy of 6 db an ablation frequency of 200 Hz. IMC images were produced using MCD viewer (V.1.0, Fluidigm) and analysis was performed using HALO (Indica labs). Tissue regions were classified using random forest with all markers included. Cells positive for each marker were manually optimized by setting a cell intensity threshold. Values for the numbers of positive cells for markers of interest were exported for analysis in GraphPad Prism V.8 (RRID:SCR_002798)

10.1136/jitc-2022-006457.supp3 Supplementary data

### Immunohistochemistry

Immunohistochemistry (IHC) was performed as previously described^40^ in the histopathology core at the CRUK CI. Briefly, tissues were removed from the mouse at the endpoint and immediately formalin-fixed for 24 hours. Fixed tissues were then processed, embedded in paraffin and sectioned (3 µm sections). Following dewaxing and rehydration, as standard, antigen retrieval was performed using Leica’s Epitope Retrieval Solution 2 (Tris EDTA) at 100°C for 20 min. Additional protein block from Dako (X090930-2) was applied. The staining using anti-mouse CD8 (Cell Signaling, #98941), anti-mouse Foxp3 (Affymetrix. #14–5773) and anti-mouse p53 (Novocastra; #NCL-L-p53-CM5p) antibodies, was performed on Leica’s automated BOND-III platform in conjunction with their Polymer Refine Detection System (DS9800) and a modified version of their standard template. Slides were dehydrated and cleared in xylene on Leica’s automated ST5020 before sections were mounted on Leica’s coverslipper, CV5030 (mounting media: DPX Mountant for histology; Sigma-Aldrich, 06 522–500 ML) and scanned using a ScanScopeAT2 (Aperio Leica Biosystems). Quantification of viable tumor tissue was performed after exclusion of necrotic area using the Halo software V.3.3.2541.405 (Indica Labs). Cell density was calculated as the number of positive cells×mm^2^ of tumor tissue analyzed. Sections of mouse spleen were used on each slide as internal control.

For the analysis of the lung metastatic burden of any individual mouse, the four right lobes and the left lobe were cut into multiple pieces and together fixed and then embedded, then treated as above. p53 staining was used for helping the detection of smaller lesions (minimum of five cells). Analysis was performed using Halo software and expressed as % of metastatic areas/total lung area analyzed. Mice with intra-abdominal/thoracic organs direct infiltration were excluded from the analysis.

### RNA sequencing

RNA was extracted from 12 s.c. allograft tumor tissues (six mice of the vehicle+isotype group or control and six mice of the AZD4635+2c5mIgG1 group or Adoi) weighing up to 30 mg. Tissues were first disrupted and homogenized using TissueLyser II and then RNA was extracted using a Qiagen RNAeasy kit, according to the manufacturer’s instructions. RNA was then quantified using a Qubit 3.0 (Life Technologies) and purity and quality were assessed using an Agilent 4150 (G2992AA) TapeStation system (Agilent). Library construction was followed by paired-end 50 bp sequencing on a NovaSeq 6000 sequencer.

### Bioinformatics analysis

Sequencing files in FASTQ format were aligned against the GRCh38 mouse genome using HISAT2 (RRID:SCR_015530) with default parameters. Samtools (RRID:SCR_002105) was used to create, index and merge BAM files of reads from different lanes belonging to individual samples. FeatureCounts (RRID:SCR_012919) was used to quantify gene-level expression of transcripts. All downstream analyses were completed in R V.4.1.2. Prior to analysis, MSI data for sequenced samples were examined. From the vehicle/isotype-treated arm (control group), sample 23,729 showed minimal necrosis, low peri-necrotic adenosine and a high ATP/AMP ratio suggesting a very high energetic state. This identified the sample as a potential outlier which was confirmed on visual inspection of a PCA (Principal Component Analysis) plot (online supplemental figure 1C). It was excluded prior to downstream analysis.

For the remaining 11 samples, initially genes were filtered to maintain only genes that were expressed at a reasonable level in >5 treatment conditions using the filterByExpr() command from the edgeR package (V.3.36.0).

Differential gene expression analyses were performed on raw read counts of the combined data object of all 11 samples. To identify significantly expressed genes between Adoi and control groups, we used a Wald test within the DESeq2 package (RRID:SCR_000154, V.1.34.0). Genes were considered differentially expressed when the analysis resulted in an adjusted p value (corrected for multiple testing using the Benjamini and Hochberg method) below 0.05. The volcano plot was generated using the EnhancedVolcano package (V.1.12.0) with the addition of custom code.

Gene set enrichment analysis of 712 genes identified as differentially expressed with p adjusted ≤0.05 and log2 (fold change) ≤−0.58 was performed via the Enrichr (RRID:SCR_001575) (online supplemental table 2) server database for Kyoto Encyclopedia of Genes and Genomes (KEGG) (https://www.genome.jp/kegg/) pathways and Gene Ontology (GO): Biological processes (http://geneontology.org/). Subsequently, enriched terms ranked for significance for each database were downloaded and are reported in online supplemental table 4 and 5. Terms of interest were selected from the top 15 ranks in each table. Genes from this study which were shown to be enriched in these terms of interest were then selected to be displayed in a heatmap. Raw counts were normalized with DESeq2 (RRID:SCR_000154) prior to visualization of gene expression levels with pheatmap (V.1.0.12). Please refer to the online supplemental methods and materials references section for all of the above.

### Analysis of human PDAC available data sets and generation of PDAC-specific adenosine signature

In order to the evaluate the correlation of the adenosine-related gene expression profile to human PDAC we analyzed 712 genes which had at least a 50% decreased expression (log2 fold change <−0.58) following adenosine inhibition treatment, of which 561 had a human ortholog (online supplemental table 2 and 3).

For the analysis of the adenosine-related gene expression in Bailey^44^ PDAC subtypes (ADEX, immunogenic, squamous and pancreatic progenitor) we derived z-scores for the 517 genes analyzed in the data set (out of the 561 genes) for the 97 patients with RNAseq data and subtype information (https://www.cbioportal.org/, RRID:SCR_014555). The z-score of all genes were summed per patient and the total number represented as the adenosine pathway gene score as previously shown^18^ and in online supplemental materials and methods.

For the generation of a PDAC-specific adenosine signature and application of this to PDAC survival, from the list of 561 human ortholog genes, we manually curated the ones-related without ambiguity to the major biological processes implicated in PDAC pathogenesis and indicated by pathway analysis (hypoxia, immunity and extracellular matrix organization). Of these genes, only those that correlated positively or negatively to survival in PDAC (https://kmplot.com)^45^ and were significantly co-expressed with CD73 and/or Adora2a in public data sets (Bailey *et al* or The Cancer Genome Atlas, TCGA) were selected. A final list of 52 genes was analyzed (online supplemental table 6).

Using PDAC specific data from TCGA^46^ available in https://www.cbioportal.org/, we derived the z-score of these 52 genes for each patient with known disease-specific survival (DSS), progression-free survival (PFS) and disease-free survival (DFS). The z-scores for all genes were summed up for each patient and were deemed high adenosine score if >0 or low adenosine score if <0, as previously shown.^18^

### Statistics

GraphPad Prism V.8 (RRID:SCR_002798) was used for statistical analyses. Analysis and comparisons of two groups was performed with two-tailed unpaired Student’s t-test when assuming Gaussian distribution or Mann-Whitney test. Analysis of three or more groups was performed with one-way analysis of variance with Tukey’s multiple comparisons post-test analysis unless otherwise specified. Kaplan-Meier analysis with log-rank Mantel-Cox test was used to evaluate difference in survivals. Differences were considered significant when p<0.05.

### Data availability statement

The data generated in this study are available within the article and its online supplemental data files or from the corresponding authors on reasonable request. Code for differential expression analysis and visualization of RNAseq data is available via GitHub https://github.com/ka-lw/AdenoPDAC.^47^

## Results

### Adenosine pathway expression on KPCY-derived cell lines

**Figure 1:**
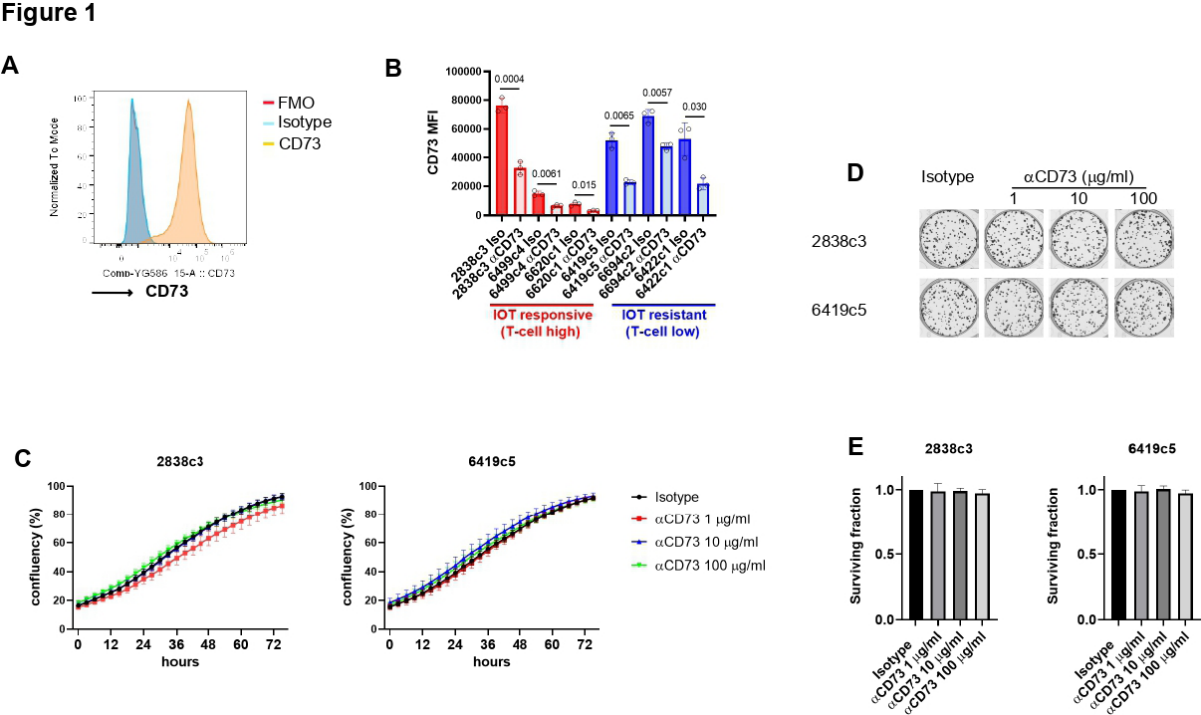
Expression of CD73 on KPCY-derived cell lines and response to anti-CD73 in vitro inhibition. (A) Representative histogram of CD73 expression on KPCY-derived cell line (2838c3) in flow cytometry. (B) CD73 expression was evaluated on KPCY-derived cell lines treated with 10 µg/mL of NIP228 (IgG isotype) or 2c5mIgG1 (anti-CD73 neutralizing antibody) for 24 hours. (C) 2838c3 (left) and 6419c5 (right) cell lines were grown with increasing concentration of anti-CD73 or isotype (100 µg/mL) and confluency was evaluated using Incucyte time lapse imaging for up to 72 hours. For each experiment, three different wells per condition were used per experiment. (D–E) Representative images (D) and graphs (E) showing survival fraction of cells (2838c3 left, 6419c5 right) from the colony-forming experiment following 8-day treatment with anti-CD73 or isotype. For each experiment, three different wells per condition were used. All data are presented as mean±SEM from experiments repeated three times. Statistical analysis was performed with two-tailed unpaired Student’s t-test (B) mixed-effect model (C) and one-way analysis of variance with post-test analysis for multiple comparisons; p values are shown in the graphs when considered significant (p<0.05). IOT, immuno-oncology therapy; FMO, fluorescence minus one; MFI, mean fluorescence intensity.

Human PDAC tumor cells express CD73 and demonstrate weak sensitivity to the targeting of CD73 in vitro.^36 48^ For this reason, we sought to investigate whether murine KPCY-derived cell lines (which are associated with contrasting ability to generate IOT-resistant or responsive tumors when reimplanted in syngeneic mice) express the proteins of the canonical adenosine pathway (CD39, CD73 and Adora2a). We found that, as in human cells, mouse PDAC cell lines express CD73 (from 72% to 99% of cells; figure 1A and online supplemental figure 2A) but demonstrate negligible or no expression of CD39 and Adora2a (online supplemental figure 2B,C). Exposing cells to an anti-CD73 antibody (2c5mIgG1) reduced significantly the detection of CD73 in all the cells after only 24-hour treatment (p<0.05 in all cell lines, figure 1B), but this did not translate into inhibition of cell growth after short or long exposure at high concentrations. In addition, confluency experiments showed that the treatment did not affect the proliferation of any of the cell lines over a period of 72 hours (figure 1C and online supplemental figure 2D,E), and colony-forming experiments performed on 2838c3 (IOT-responsive) and 6419c5 (IOT-resistant) demonstrated no differences in terms of number or size of the colonies formed after 8 days of continuous treatment (figure 1D,E and online supplemental figure 2F). Accordingly, in vitro inhibition of CD73 was performed on human PDAC cell line PANC-1 using anti-human CD73 oleclumab (online supplemental figure 2G-I). Oleclumab did not reduce tumor cell growth when used alone or in combination with multiple cytotoxic agents, either as a concomitant treatment (online supplemental figure 2G) or when used as a pretreatment (online supplemental figure 2H,I). In order to evaluate whether a direct effect of anti-CD73 exposure affects cell proliferation, reducing adenosine formation, we cultured KPCY-derived cell lines with increasing concentrations of AMP and 5’-N-(Ethylcarboxamido)adenosine (a stable form of adenosine). We were able to demonstrate that adenosine and AMP have no effect on the proliferation capacity of these cell lines (online supplemental figure 2G,H), corroborating the hypothesis of a non-cancer cell direct effect of anti-CD73 therapeutic approaches.

### Comparison between KPCY-derived cell lines allograft and KPC autochthonous tumors

Despite the lack of activity in our cell line experiments, preliminary responses have been reported in early phase clinical trials when quemliclustat (a CD73 small molecule inhibitor) or oleclumab were combined with PD-(L)1 [Programmed cell death-(ligand) 1] inhibition and standard-of-care therapy.^49–51^ As we hypothesized that this might be the result of impact on the TME, we investigated the expression of this pathway on the tumor-infiltrating immune cells for different murine PDAC models, including KPCY-derived cell line allografts (with differential response to IOT) and autochthonous KPC tumors. In order to understand the complexity and similarities of the immune system in these models we first compared the immune infiltration of the cell line allografts to the KPC model.

The immunosuppressive characteristics identified in autochthonous KPC tumors, appear to be more aligned with those of the IOT-resistant model. In particular regarding lymphocyte populations, KPC tumors are usually infiltrated by a low number of CD8^+^ T cells (mean number of CD45^+^ cells for 2838c3 is 6.9%, 0.9% for 6419c5 and 1.9% for KPC tumors), which express lower levels of PD-1, a known marker of activation/exhaustion (mean 70% vs 13% vs 10%) as shown in online supplemental figure 3A and have a similar total CD4^+^ T cells (online supplemental figure 3C) and regulatory T-cell (Tregs) infiltration (mean 3.2% vs 1% vs 0.7%, online supplemental figure 3B) to IOT-resistant tumors. Moreover, KPC tumors showed greater heterogeneity regarding myeloid infiltrating populations (online supplemental figure 3D–H). These results suggest that our IOT-resistant and responsive models stand out as the extreme clonotypes which can arise from the complex and heterogeneous biology found in KPC autochthonous tumors.

### The adenosine pathway is enriched in immune cells infiltrating PDAC models

**Figure 2:**
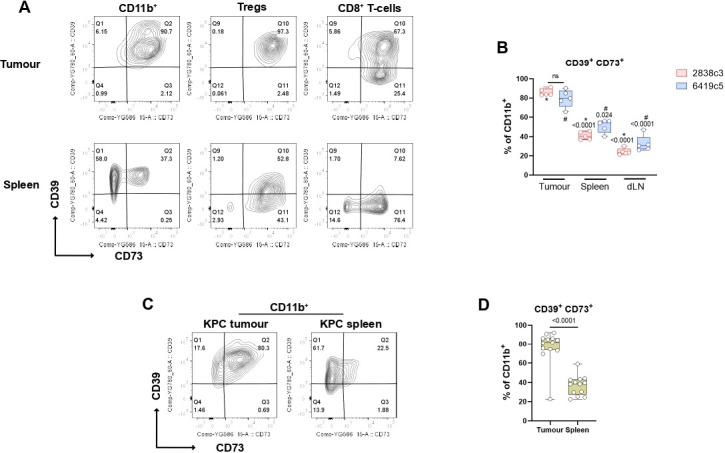
The adenosine pathway members are expressed on PDAC-infiltrating immune cells. (A) Representative flow cytometry plots showing expression of CD39 and CD73 on myeloid population (left), Tregs (middle) and CD8^+^ T cells (right) from KPCY-cell line derived tumor (upper) and matched spleen (lower) (N=5 mice per group). (B) Box and whisker graph showing CD39^+^CD73^+^ double expression on CD11b^+^ cells for 2838c3 (N=5) and 6419c5 (N=5) in tumors, matched spleens and tumor draining lymph nodes. (C–D) Representative flow cytometry plots (C) and box and whisker graph (D) showing CD39^+^CD73^+^ double expression on CD11b^+^ cells infiltrating autochthonous KPC tumors (N=13). All data are presented as interleaved box and whiskers. Statistical analysis was performed using one-way analysis of variance with post hoc test analysis for multiple comparisons (B) and two-tailed unpaired Student’s t-test (D) p values are shown in the graphs when considered significant (p<0.05). PDAC, pancreatic ductal adenocarcinoma; Tregs, regulatory T cells; dLN, draining lymph node.

We hypothesized that the adenosine pathway might have a more impactful role in the TME, as opposed to a cell autonomous effect. For this reason, we investigated the expression of the adenosine pathway components on tumor-infiltrating immune cells which represent a significant proportion of cells seen in PDAC lesions. We showed a highly significant enrichment in both the IOT-resistant and IOT-responsive models for CD39^+^CD73^+^ double-expressing immune cells, when compared with secondary lymphoid organs (spleen and nodes). In particular, the majority (65–91% in tumor vs 36–56% in spleen) of tumor-infiltrating CD11b^+^ myeloid cells express the two receptors, due to an increase in expression of CD73, given that those cells are normally CD39^+^ (figure 2A,B). Similar results were shown for Tregs and CD8^+^ T cells, which are normally CD73^+^ and displayed an increase in expression of CD39 in tumor, compared with the secondary lymphoid organs counterparts (online supplemental figure 4A; p<0.05). There was no significant difference in these findings when comparing the two models, despite their differential response to IOT. We then confirmed these findings in KPC autochthonous tumors and found similar results, with a significant increase of CD39/CD73 double expressing CD11b^+^ myeloid cells infiltrating the tumors compared with spleens (mean 77% vs 36% p<0.0001, figure 2C,D), harvested from the same mice. Of note, four KPC mice had synchronous metastases (three liver and one spleen), and in three of these, myeloid cells infiltrating the metastases were also enriched for CD73^+^CD39^+^ double expression (online supplemental figure 4C) when compared with secondary lymphoid organs. Significant increased co-expression was also observed for Tregs and CD8^+^ T cells in KPC tumors when compared with mesenteric or inguinal lymph nodes (online supplemental figure 4B). An increased percentage of CD39/CD73 double expressing CD8 T cells and Tregs was noted in the spleens (online supplemental figure 4B) if compared with what was found in non-tumor bearing mice (not shown) or s.c. tumor bearing mice, suggesting trafficking of immune lymphoid populations between primary lesions and closer lymphoid organs.

### Adenosine distributes primarily in the hypoxic areas surrounding necrosis

**Figure 3:**
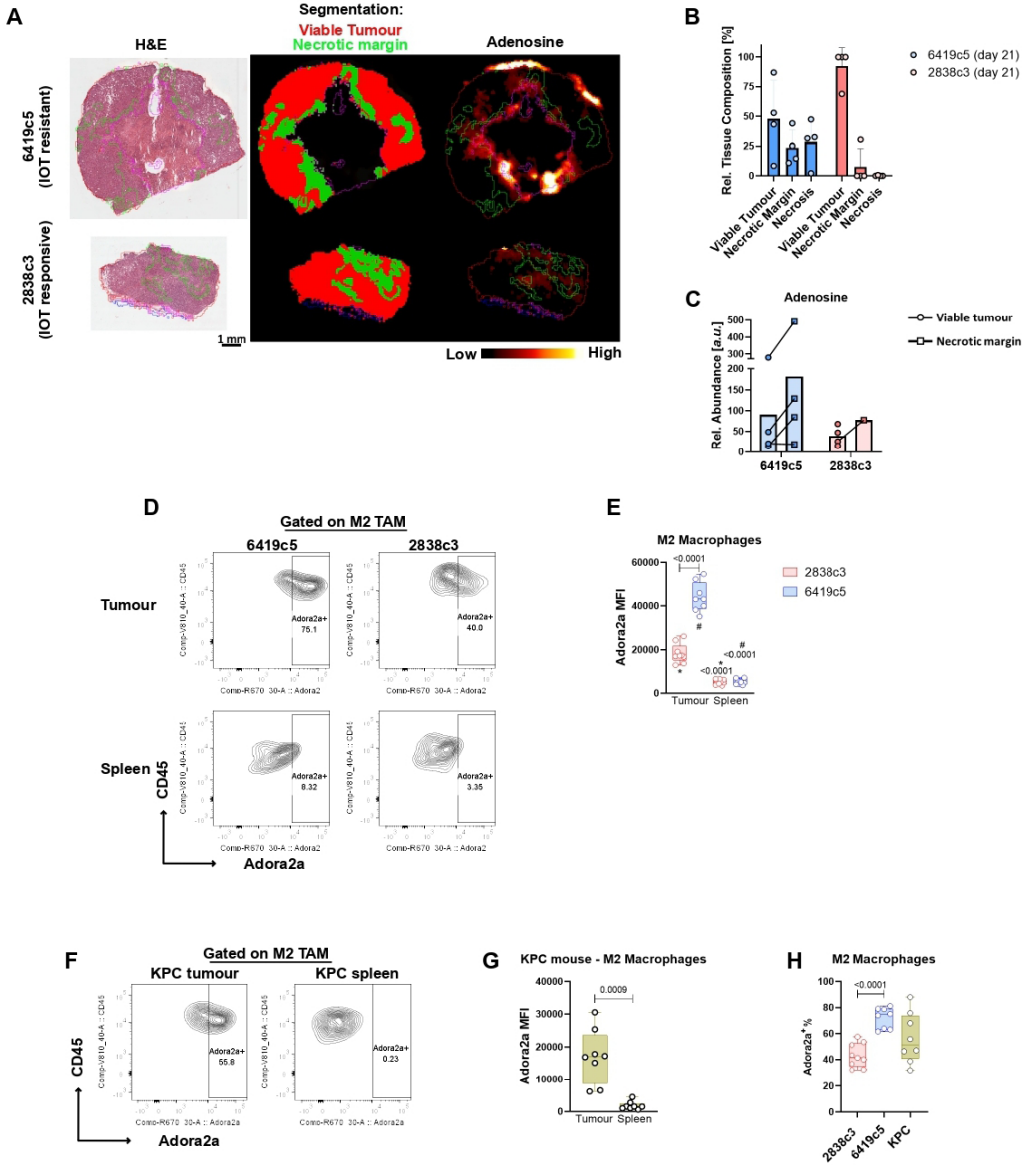
Adenosine distribution is spatially heterogeneous and targets myeloid subpopulations. (A) Mass spectrometry imaging (MSI) representative images showing adenosine expression and distribution in pancreatic ductal adenocarcinoma subcutaneous allografts (four mice per group) at day 21 post-implantation. Classification was obtained based on metabolites expression and is represented as follows: viable tumor (red), necrotic margins (green). (B) Relative tissue composition differences of viable tumor, necrotic margin and necrotic areas for 6419c5 (N=4) and 2838c3 (N=4) allografts at day 21 post-implantation. As shown, necrosis was present in only one 2838c3 sample at 21 days post-implantation. Error bars represent SD. (C) MSI analysis showing relative abundance of adenosine in the different areas in 6419c5 and 2838c3 allografts at day 21 post-implantation. Bars represent means. (D–E) Representative plots (E) and summary graph (F) from flow cytometry analysis showing Adora2a expression on protumorigenic M2 macrophages in allografts (upper) derived from 6419c5 (left) and 2838c3 (right) implantation. Same expression is shown in M2 macrophages in matched spleens (lower) (8–9 mice per group were used). (F–G) Flow cytometry plots (G) and graph (H) showing expression of Adora2a in M2 macrophages comparing KPC (n=8) autochthonous tumors and matched spleens. (H) Box and whisker plot of the percentage of M2 macrophages positive for Adora2a comparing two allografts (6419c5 and 2838c3) and KPC tumors. Statistical analysis was performed using one-way analysis of variance with post hoc test analysis for multiple comparisons (E,H) and two-tailed unpaired Student’s t-test (G) p values are shown in the graphs when considered significant (p<0.05). IOT, immuno-oncology therapy; TAM, tumor associated macrophage; MFI, mean fluorescence intensity.

Given the enrichment of the adenosine pathway in the TME of PDAC models, we anticipated that eAdo might have been abundant in the TME. Using MSI we evaluated the presence and the distribution of the purinergic system in the TME of IOT-resistant tumors. The tissue classification and segmentation approaches were driven by tissue-defining metabolic patterns. Areas characterized by a high energetic state defined by a high abundance of ATP and ADP and a low abundance of depleted high energy phosphates such as AMP, were called *viable tumor*. In contrast, areas of tumor adjacent to necrosis (termed *necrotic margin*) were characterized by high abundance of lactate, products of ribonucleotide catabolism (ie, xanthine and hypoxanthine) and other metabolites associated with tissue hypoxia and an overall energy-deprived state (figure 3A,B). We found that adenosine is present in high concentrations in the microenvironment of PDAC murine models, although showing a heterogenous distribution, with high abundance in the hypoxic necrotic margin areas (figure 3C and online supplemental figure 4D). The IOT-resistant model 6419c5 shows a higher abundance of adenosine, mostly due to the paucity of necrotic areas in this 2838c3 IOT-responsive model (figure 3A–C). This trend of adenosine expression was then evaluated in other KPCY-derived allografts, confirming the lower abundance of adenosine in the IOT-responsive models and higher expression in one other IOT-resistant tumor (6694c2), particularly in the *necrotic margin*s. A third IOT-resistant model (6422c1) showed levels of adenosine similar to the IOT-responsive tumors, suggesting that the expression of the adenosine pathway might not be a unique feature in the generation of immunosuppression in murine PDAC (online supplemental figure 4E,F). The autochthonous KPC tumor model showed a higher degree of complexity in their histological composition (online supplemental figure 4G). MSI analysis demonstrated a primarily hypoxia-driven metabolic phenotype in which adenosine and other metabolites such as succinate, lactate and metabolites associated with purine metabolism were elevated. The hypoxic phenotype, and therefore adenosine, was not limited to the margin around established necrosis, but found throughout the samples independent of established necrosis. This limited the ability to delineate the necrotic margin as it was possible in the KPCY-derived s.c. tumors, but confirmed the importance of hypoxia in the generation of adenosine. When we investigated the cell population distribution in the different areas using IMC in IOT-resistant tumors, we noted in the necrotic margin areas a 2.7-fold increase in the number of infiltrating CD11b^+^ myeloid cells (mean 1970 in the *necrotic margin* vs 730 in *viable tumor* CD11b^+^ cells/mm^2^), that led to a significant decrease of the ratio between cancer cells/myeloid cells (online supplemental figure 5A). This again suggests that myeloid cells have an instrumental role in the generation of adenosine in this aggressive model of PDAC.

### Expression of ADORA2A receptor on myeloid subpopulations of pancreatic cancer models

Having shown in these models that in the PDAC microenvironment, immunosuppressive adenosine is present abundantly, we then investigated which cells within the microenvironment might be responsive to this. We investigated the expression of the adenosine A2a receptor (Adora2a, the receptor with the highest affinity for adenosine) that has been found to be frequently overexpressed in human tumors, by flow cytometry. We found that Adora2a was highly expressed by tumor-infiltrating myeloid population when compared with the spleen (online supplemental figure 5B,C) and this expression was significantly higher in the IOT-resistant model in terms of MFI (mean fluorescence intensity 10,000 vs 6700, p<0.0001) and % of Adora2a^+^ myeloid cells (15% vs 11%, p=0.009). In contrast, lymphoid populations infiltrating the tumors were negative for Adora2a expression (online supplemental figure 5B, bottom). When comparing different subpopulations, protumorigenic M2 macrophages, infiltrating both IOT-resistant and IOT-responsive PDAC showed high positivity for the receptor. The IOT-resistant model had higher expression of Adora2a compared with the IOT-responsive model (figure 3D,E; p<0.0001) and percentage of Adora2a^+^ M2 positivity (72% vs 43%; p<0.0001) (figure 3H). Once more, these findings were confirmed in KPC tumors where Adora2a was found to be increased in M2 macrophages infiltrating the lesions when compared with matched spleens (figure 3F,G). The KPC model demonstrated once again the heterogeneity of pancreatic lesions, which in terms of M2 macrophages, positive for the Adora2a receptor, covers the entire range of expression seen in the two s.c. models used (figure 3H). Notably, of three KPC mice where metastatic nodules were found, Adora2a expression was found retained in the M2 macrophages infiltrating the secondary lesions (online supplemental figure 5D, top panel). Lymphoid cells were negative for Adora2a both in tumors and in metastases (online supplemental figure 5D, bottom panel).

In addition to protumorigenic macrophages, Adora2a expression was found enriched in other myeloid immune populations infiltrating the tumors. In particular CD11b^−^ DCs, CD11b^+^ DCs (online supplemental figure 5E), M1 macrophages (online supplemental figure 5F), and monocytic MDSCs (mo-MDSCs) (online supplemental figure 5G) for both models and granulocytic MDSCs (gMDSCs) for IOT-resistant tumors (online supplemental figure 5H) express significantly higher Adora2a amount when compared with matched spleens. This expression differs significantly between IOT-responsive and resistant models in CD11b^+^ DCs (mean MFI 2080±174 vs 3190±636, respectively; p=0.007), M1 macrophages (4520±983 vs 6940±1690; p=0.02) and mo-MDSCs (1890±479 vs 6110±1870; p=0.001) (online supplemental figure 5E–H).

### Targeting adenosine pathway delays tumor growth of murine pancreatic cancer representing a combinational therapeutic opportunity

**Figure 4:**
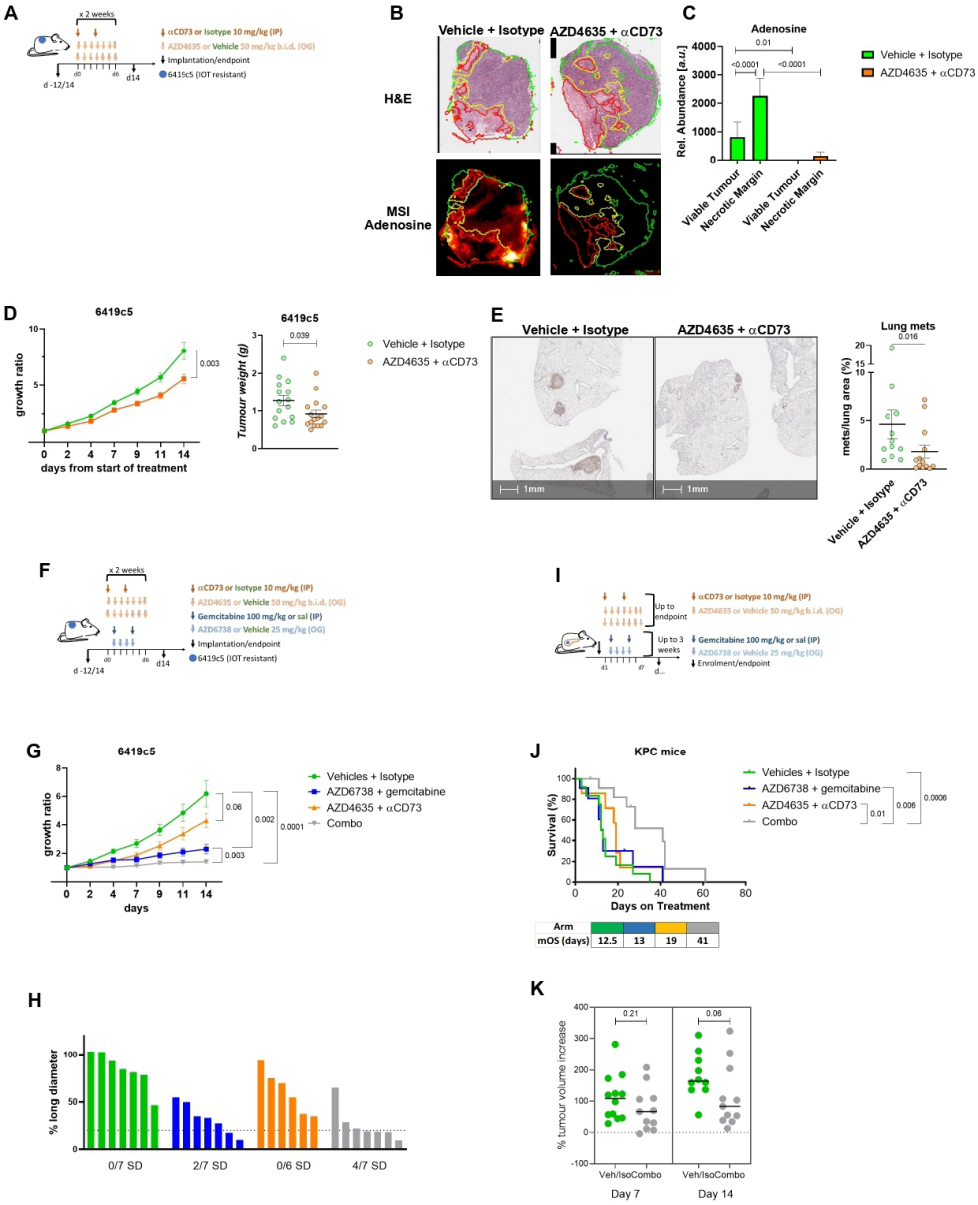
In vivo modulation of the adenosine pathway reduces tumor growth and metastasis and improves the efficacy of cytotoxic treatment. (A) Schedule of adenosine inhibition (Adoi) treatment. Treatment was started following 12–14 days from implantation and continued for 2 weeks. Antibody anti-CD73 (2c5mIgG1, murine IgG1) was dosed two times per week intraperitoneally at 10 mg/kg. Adora2a inhibitor (AZD4635) was given by oral gavage two times a day at 50 mg/kg. (B–C) Mass spectrometry imaging (MSI) representative images (B) and MSI analysis graph with relative abundance (a.u.) (C) showing adenosine expression and distribution in PDAC allografts treated with vehicle+isotype or Adoi, at day 14 from treatment start (six mice per treatment group). Classification was obtained based on metabolites expression and is represented as follows: viable tumor (green line), necrotic margins (yellow line). (D) Tumor growth ratio (left) and weight (right) of 6419c5 allografts in C57Bl/6 mice treated with anti-CD73+AZD4635 (N=16) or vehicle+isotype (N=15). (E) Representative image (left) and graph (right) showing % of area of the lung analyzed occupied by metastasis (met/lung areas×100) in vehicle+isotype (N=12) and anti-CD73+AZD4635 (N=13), evaluated for the presence of spontaneous occurrence of lung metastases. Every dot represents a single mouse. Tissues were stained with an anti-p53 antibody to highlight the presence of cancer cells. A group of more than five p53-positive cells was counted as metastasis. (F) Schedule of 6419c5 tumor allografts 14-day treatment as following (N=7 mice per group): vehicles+isotype, AZD6738+gemcitabine, anti-CD73+AZD4635, AZD6738+gemcitabine + anti-CD73+AZD4635. (G) Tumor growth ratio (14 days) of 6419c5 tumor allografts treated as above (H) percentage change in the long diameter length following 14 days of treatment per group. Number of mice with stable disease (SD, <20% increase and <30% decrease) are shown at the bottom. (I) Schedule of KPC mice treatment as following: vehicles+isotype (12 mice), AZD6738+gemcitabine (11 mice), anti-CD73+AZD4635 (7 mice), AZD6738+gemcitabine + anti-CD73+AZD4635 (combo, 12 mice). Adenosine inhibition was administered until endpoint, while AZD6738+gemcitabine was allowed up to 3 weeks. (J) Survival analysis of KPC mice treated as above, including median overall survival (mOS). (K) Percentage of tumor volume increased in vehicles versus combo group at day 7 and 14 of treatment. All data are presented as mean±SEM. Statistical analysis was performed using Mann-Whitney test (D–E,K) mixed-effect model (F) and one-way analysis of variance with post hoc test analysis for multiple comparisons (C). Log-rank Mantel-Cox test to evaluate difference in survivals (J) p values are shown in the graphs and considered significant when p<0.05. IOT, immuno-oncology therapy; IP, intraperitoneal.

Our data suggest a mechanism by which the myeloid population contributes to the protumorigenic functionality of the pancreatic cancer microenvironment, where eAdo generated by the myeloid cell populations and cancer cells would target and stimulate further the myeloid cell subpopulations, in particular the protumorigenic M2 macrophages. Therefore, we inhibited in vivo eAdo formation and function, using an antibody against CD73 (2c5mIgG1) and a small molecule inhibitor of Adora2a (AZD4635), a combination (Adoi) which would maximize the inhibition of the axis. The 14-day treatment was started after the microenvironment was allowed to establish (12–14 days after implantation) in the IOT-resistant allografts (figure 4A). The anti-CD73 was extremely effective in reducing the expression of CD73 on the surface of all live cells (online supplemental figure 6A). MSI data confirmed marked reduction in adenosine formation in the TME (figure 4B,C). In particular, adenosine was completely abolished in the viable tumor areas, while a small amount remained in the necrotic margins, accounting for a 95% decrease (figure 4C), highlighting the importance to block residual adenosinergic signaling downstream CD73 inhibition, co-targeting adenosine receptors. The effectiveness of the treatment on the extracellular purinergic pathway was also supported by the decrease of molecules downstream of adenosine (adenine and inosine in viable tumor and necrotic margin areas) (online supplemental figure 6B,C), and the increase of upstream and alternative pathway molecules as AMP (in the necrotic margin, online supplemental figure 6D) and xanthine (in both viable tumor and necrotic margin, online supplemental figure 6E) respectively. There was no change in the distribution of ATP, ADP and hypoxanthine (online supplemental figure 6F–H).

The Adoi approach led to a 30% reduction of tumor growth ratio (mean of 8 vs 5.5-folds increase from the baseline, p=0.003, figure 4D and online supplemental figure 7A) and tumor weight (median 1.24 vs 0.78 g, 0.039 figure 4A). Single agent AZD4635 induced a similar tumor control to the full combination. However, the therapeutic effect was delayed (online supplemental figure 7B), with a 30% growth reduction in the combination arm when compared with Adora2ai inhibition alone with single agent AZD4635, over the first 4 days of treatment (p=0.01). For biological reasons, given also that AZD4635 is a competitive inhibitor and its efficacy is dependent on the amount of eAdo, we chose to use it in combination with anti-CD73 antibody to maximize the blocking on eAdo effects. These data support previous findings showing the same combination had greater antitumor immune effect.^39^ In contrast, targeting the adenosine pathway with Adoi in 2838c3 allografts where necrosis and consequently peri-necrotic adenosine are low, and M2 macrophages express lower level of Adora2a, did not translate into tumor growth reduction (online supplemental figure 7C). The adenosine pathway has been shown to control the metastatic process and several authors have shown that inhibiting this axis can reduce the metastatic burden in mouse models.^52 53^ However, we were able to show for the first time that blocking adenosine generation and function can significantly reduce the occurrence of spontaneous metastasis in an IOT-resistant preclinical model of PDAC. The 6419c5 s.c. model spontaneously develops lung metastases in 100% of the mice and blocking adenosine strongly reduced the metastatic burden (median of % mets/lung area 0.77% vs 2.6%; p=0.016; figure 4E).

These data suggest that targeting myeloid related, eAdo formation and effect would have an effect on tumor growth, making this approach a candidate for combinatorial therapeutic studies. Indeed, when combined with cytotoxic treatment (AZD6738, an ATR inhibitor and gemcitabine; figure 4F,G) or IOT [anti-CD40 agonist (F), anti-CTLA-4 (C) and anti-PD-L1 (P),FCP; online supplemental figure 7D], the adenosine modulation reduced further the tumor growth rate of the aggressive IOT-resistant 6419c5 tumor model. AZD6738/gemcitabine alone was able to significantly slow the growth of the IOT-resistant model (2/7 SD (28.5%), but the addition of the adenosine blocking (AZD4635+2c5mIgG1) led to further stabilization of the tumor growth in a 2-week regimen (p=0.003 vs AZD6738/gem alone; 4/7 SD (57.1%); figure 4G,H). These data supported the investigation of the same combination in autochthonous tumors in KPC mice (figure 4I) to assess whether the addition of Adoi to cytotoxic treatment (AZD6738+gemcitabine) prolonged survival in this model, considered a gold standard in this disease. As figure 4J shows, the combination of Adoi and ATRi/gem induced a threefold increase in median overall survival (mOS) in KPC mice compared with control groups (vehicles+isotype 12.5 days vs combo 41 days, p=0.0006). The four-drug regimen is significantly better than single schedule arms (mOS: Adoi 19 days and AZD6738+gemcitabine 13 days). A weekly ultrasound revealed a tendency for tumor stabilization in mice treated with cytotoxic therapeutics plus adenosine inhibition, when compared with the control arm during the first 2 weeks of treatment (figure 4K).

Combining adenosine blockade (Adoi) and immunotherapy (FCP) reduced significantly tumor growth of the IOT-resistant allograft model when compared with control treatment (65% tumor growth reduction; p=0.002) and FCP (35% tumor growth reduction; p=0.021) arms (online supplemental figure 7D–F). Data from two separate experiments with the same controls showed that adding Adoi to FCP remains the best combination in controlling tumor growth (online supplemental figure 7G–I). These data suggest again that targeting the adenosine pathway in PDAC offers a new strategy to modulate the antitumor immune response.

### Targeting the adenosine pathway results in reprogramming of the Tme in the IOT-resistant pancreatic cancer mouse model

To understand the role of adenosine modulation in reprogramming the TME, given the effect on tumor growth alone or in combination we analyzed the changes in immune infiltration following anti-CD73 and AZD4635 treatment.

**Figure 5:**
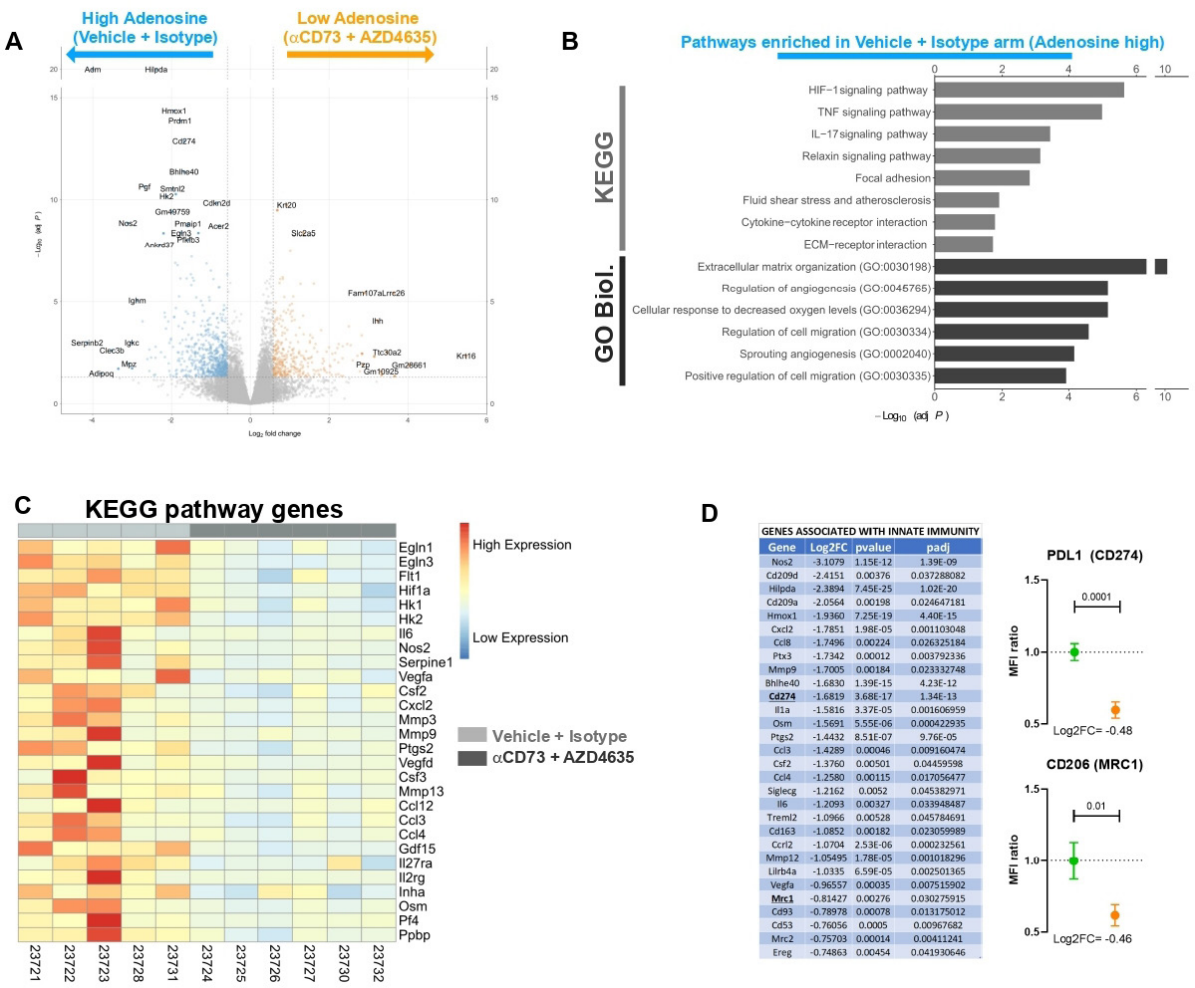
Adenosine inhibition remodels the pancreatic ductal adenocarcinoma tumor microenvironment. (A) Volcano plot related to 6419c5 allografts. Genes overexpressed in vehicle+isotype arm are on the left side (blue dots), and genes overexpressed in the anti-CD73+AZD4635 arm are on the right side (Adoi, orange dots). (B) Enrichment bar plot of significantly overexpressed pathways in the vehicle+isotype group (adenosine high), according to KEGG and GO biological processes. (C) Heatmap showing genes regulated during treatment (light gray for controls, dark gray for Adoi) which are part of the significantly different pathways according to KEGG. (D) Table (left) showing genes associated with innate immunity that are downregulated by Adoi treatment. Validation of RNAseq data (right graphs) through flow analysis of PD-L1 and CD206 expression (MFI ratio) on intratumoral live cells (8 mice in the control and 10 in the Adoi arms). The samples analyzed for validation were from different mice from the ones used for RNAseq. GO, Gene Ontology; KEGG, Kyoto Encyclopedia of Genes and Genomes; RNAseq, RNA sequencing; PDL1, Programmed death-ligand 1; MFI; mean fluorescence intensity; ECM, extracellular matrix.

The TME of PDAC is an intricate structure that relies on the presence of multiple non-malignant cells. This TME is recapitulated in preclinical models of PDAC.^38^ In order to investigate the broader effect of Adoi in the 6419c5 PDAC model, given the effect we have seen on tumor growth when used alone or in combination, we performed a bulk RNAseq analysis of the 6419c5 model treated with Adoi or control (five vs six mice, see methods). Following the 14-day treatment with AZD4635 and anti-CD73 the relative expression of 712 genes decreased by ≥50% (log fold <−0.58; figure 5A). KEGG and GO biological process pathway analysis revealed that eAdo has a broad impact on TME of the IOT-resistant model of PDAC (figure 5B and online supplemental figure 8A). Genes associated with hypoxia response and vasculogenesis (eg, *Hif1a*, *Slc2a1*, *Hilpda*, *Adm*, *Vegfa*, *Vegfd*), immunity and immune suppression (eg, *Cd274*, *Cd209*, *Mrc1*, *Cd200*, *Il1a*, *Il6*, *Ptgs2*) and tumor stroma/extracellular matrix (ECM) organization (eg, *Col5a3*, *Col6a3*, *Itga2*, *Mmp13*, *Mmp3*, *Mmp9*, *Ereg*, *Pthlh*) were significantly downregulated by the treatment (figure 5B,C).

Of note, several functional and structural downregulated genes were associated with the innate immune system (figure 5D left), particularly with M2 polarized macrophages (eg, *Cd209*, *Mrc1*, *Mrc2*, *CD163*). RNAseq analysis also showed a significant decrease of the expression of *Cd274* (PD-L1) gene, suggesting a strong rationale for the use of adenosine inhibition as combination for immunotherapy studies involving immune checkpoint inhibitors. The downregulation of *Mrc1* (CD206) and *Cd274* (PD-L1) was validated analyzing their protein expression on tumor-infiltrating live cells following Adoi treatment. As shown in figure 5D (right), the proteins of these genes were strongly downregulated in the adenosine inhibition arm (38% and 41% reduction of CD206 and PD-L1 on live cells respectively).

Furthermore, the adenosine signaling has previously been associated with the presence of hypoxia^54 55^; here we show for the first time that the presence of a functional eAdo pathway is responsible for the expression of several genes related to hypoxia (including *Hif1a*) suggesting the presence of a positive feedback loop.

**Figure 6:**
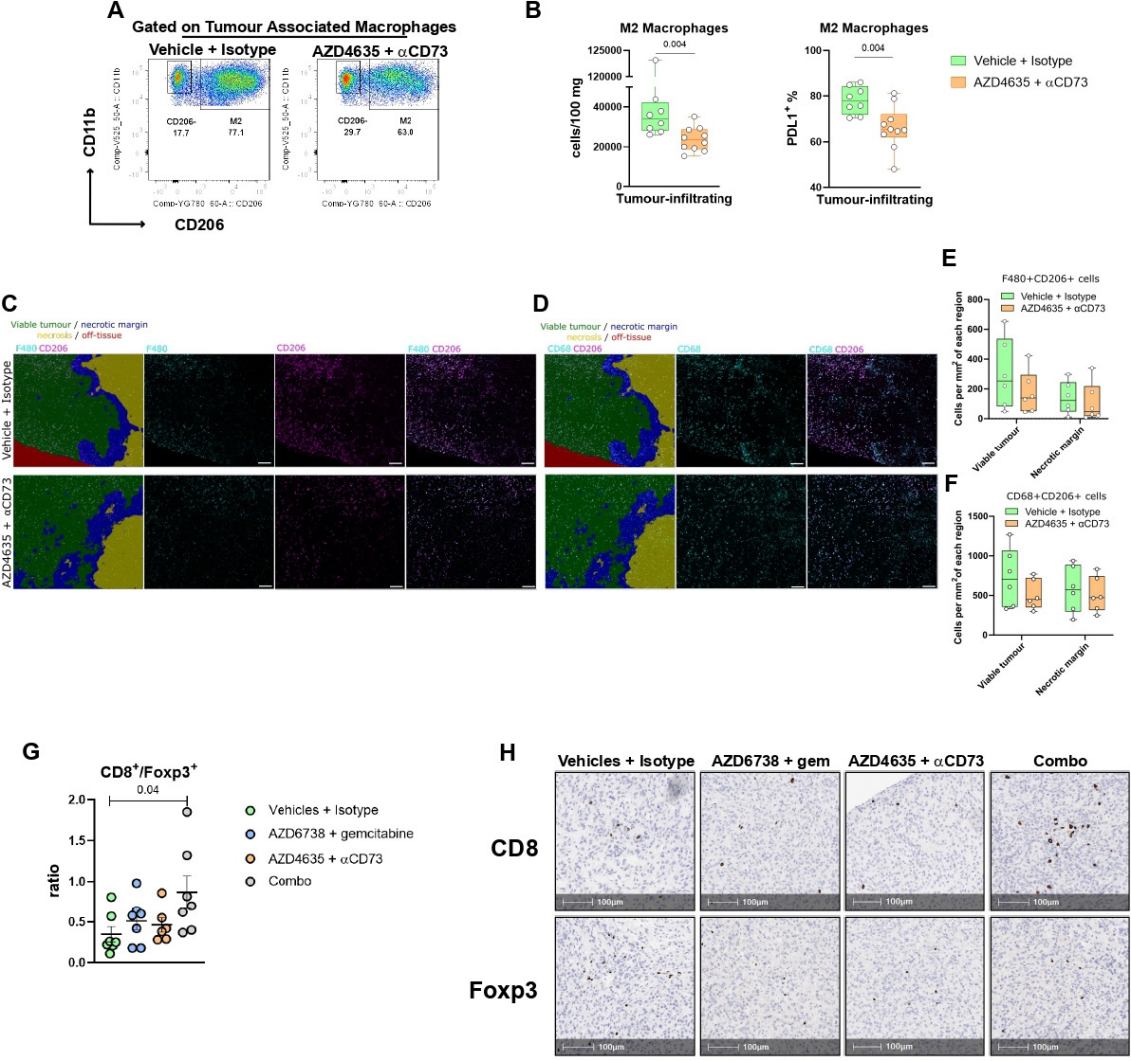
Immunosuppressive immune subpopulations are modulated following adenosine pathway blockade. (A) Representative flow cytometry plots showing tumor associated macrophages infiltrating 6419c5 allografts following 14 days of treatment with vehicle+isotype (left panel) or anti-CD73+AZD4635 (right panel). (B) M2 macrophage allograft infiltration (left, number of cells/100 mg of tumor) and (right) percentage of M2 macrophages positive for PD-L1 (8 and 10 mice per group analyzed). (C–D) Representative IMC image of F4/80 and CD206 (C) and CD68 and CD206 (D) positive cells infiltrating a 6419c5 allograft merged (first panel of C and D) or not with segmentation following Adoi (lower panels) or control (upper). The tissue segmentation of the IMC image was performed by Random Forest Classification using all markers analyzed. The scale bar on the IMC image is 200 µm. Segmentation shows viable tumor (green), necrosis (yellow), necrotic margin (blue) and off-tissue (red). (E–F) The bar plots show cell density (number of cells per mm^2^) of F480^+^CD206^+^ cells (E) and CD68^+^CD206^+^ (F) per segment area (six mice per treatment group). (G–H) Immunohistochemistry analysis showing 6419c5 tumor-infiltrating CD8^+^/Foxp3^+^ ratio (G) at day 14 of the following treatments: Vehicles+isotype (N=7), AZD6738+gemcitabine (N=7), aCD73+AZD4635 (N=6) and AZD6738+gemcitabine + aCD73+AZD4635 (N=7). (H) Representative immunohistochemistry images of the latter. All data are represented as box and whisker plots. Statistical analysis was performed using Mann-Whitney test (B–H) and one-way analysis of variance with post hoc test analysis for multiple comparisons (E–H) p values are shown in the graphs when considered significant (p<0.05). IMC, imaging mass cytometry; PD-L1, Programmed death-ligand 1.

Considering that the RNAseq analysis indicated that the innate immune system was affected by adenosine inhibition, we analyzed the changes in immune infiltration following anti-CD73 and AZD4635 treatment. Flow cytometry analysis confirmed the findings highlighted by the RNAseq analysis. Following treatment, tumors were less likely to be infiltrated by M2 macrophages (median approximately 35,000 vs 23,000 cells/100 mg; p=0.004; figure 6A,B), in particular PD-L1^+^ ones (median 79% vs 65%; p=0.004; figure 6B, right). IMC analysis also revealed a trend towards reduction of M2 macrophages following treatment (F4/80^+^ CD206^+^ (figure 6C–E) or CD68^+^ CD206^+^ (figure 6D–F)) and the trend was more pronounced in viable tumor compared with necrotic margin, but the difference was not significant. Notably this reduced trend in infiltration is present in areas other than those where adenosine is abundant, suggesting that adenosine could be stimulating the production of factors affecting recruitment of macrophages in the viable tumor areas. Blocking the axis, also led to a reduced frequency of infiltrating Tregs (mean 42% vs 27%; p=0.03, online supplemental figure 8B,C) and a reduction of PD-L1 expression for all live cells within the tumor, more prominently CD45^+^ cells (p=0.008) as shown in online supplemental figure 8D. However, the expression of PD-L1 declined in F4/80^+^ macrophages (p=0.02) but not in DCs following treatment (online supplemental figure 8E). Of note, the combination of 2c5mIgG1 and AZD4635 is required to reduce the M2 macrophage infiltration (p=0.03, online supplemental figure 8F) and the ratio of M2/M1 macrophages (p=0.04; online supplemental figure 8G) in the tumor, while single agents fail to do so. Finally, IMC data also showed a trend towards a reduction (not significant, ns) of total macrophages in the treated tumors as shown by flow cytometry, again more evident in the viable tumor regions (figure 6C,D and F4/80 and CD68 panels and online supplemental figure 8H). IMC also showed a trend towards global reduction (ns) of M1 macrophages (F4/80^+^ CD206^–^ MHC-II^+^) (online supplemental figure 8I,J) which was not apparent in the flow analysis. Further studies would shed light on the relevance of these results, analyzing the contribution of adenosine abundance and spatial distribution to modeling of tumor infiltration by distinct macrophage subpopulations beyond M1-M2 dichotomy.

Considering the reduction of the immune suppression in the TME following Adoi, we explored changes associated with the adaptive immune system following combination of adenosine inhibition and cytotoxic or immunotherapy therapeutics. To assess whether the combination of Adoi plus ATRi/gemcitabine had an effect on the immune infiltration of the TME of IOT-resistant model (6419c5 allografts), we performed IHC staining for CD8 and Foxp3 and showed that the quadruple combination almost tripled the ratio CD8/Tregs (median 0.70 vs 0.25 of vehicles+isotype group; p=0.04; figure 6G,H). The four-drug combination produced an enhanced increase of the CD8^+^ cell infiltration (median 4.5 cells/mm^2^ vs 2.7 in the control group, 3.9 in the AZD6738/gem group and 2.8 in the AZD4635/αCD73 group) and reduction of Foxp3^+^ cell infiltration (median 6.5 cells/mm^2^ vs 8.1 in the control group, 9.3 in the AZD6738/gem group and 7.2 in the AZD4635/αCD73 group) (online supplemental figure 8K). Similarly, combination with IOT (Adoi+FCP) determined a further increase of the intratumoral CD8/Tregs ratio induced by FCP treatment (ratio means control 0.28 vs FCP/Adoi 6.10 p=0.008; online supplemental figure 8L) mostly due to the repression of the Treg recruitment induced by IOT alone.

### Adenosine-related gene expression is associated with phenotype and survival in human pancreatic cancer

**Figure 7:**
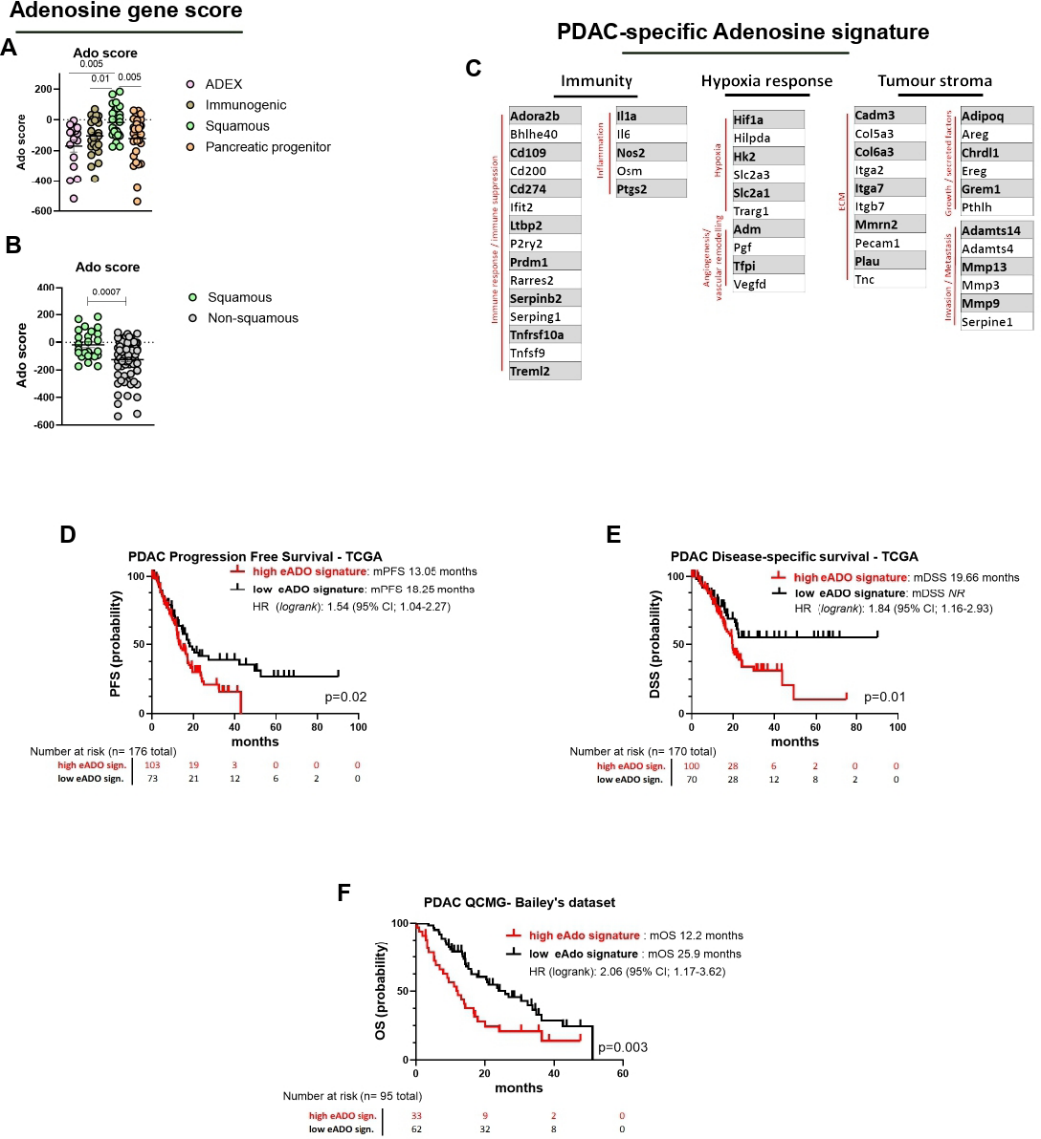
RNA gene expression demonstrates a crucial role for the adenosine pathway in correlation to human PDAC prognosis. (A–B) Human ortholog genes with a 50% downregulation following 14 days of Adoi, were scored using z-score derived from Bailey’s^44^ subtypes data set. Genes scores comparing Adex, immunogenic, squamous and pancreatic progenitor (A) or squamous versus non-squamous (B) are shown. Each dot represents a single patient. (C) PDAC-specific gene signature of 52 genes used for analysis of the TCGA human PDAC data set, related to the main pathways implied (immunity, hypoxia response and tumor stroma). (D–E) Gene signature applied to PDAC TCGA data set (D). Kaplan-Meier curves show progression-free survival (PFS, 176 patients) and (E) disease-specific survival (DSS, 170 patients). (F) Gene signature validation applied to PDAC QCMG Bailey’s data set. Kaplan-Meier curve shows overall survival (OS, 95 patients). Three genes (*Pecam1*, *Trarg1* and *Vegfd*) were *not assessed* in Bailey’s data set, thus the signature is composed of 49 genes. Data in A–B are presented as mean±SEM. Statistical analysis was performed using one-way analysis of variance with post hoc test analysis for multiple comparisons (A) two-tailed unpaired Student’s t-test (B) and log-rank Mantel-Cox test to evaluate difference in survivals (D–F) p values are shown in the graphs when considered significant (p<0.05). eAdo, extracellular adenosine; mOS, median overall survival; PDAC, pancreatic ductal adenocarcinoma; mPFS, median progression-free survival; TCGA, The Cancer Genome Atlas; mDSS, median disease-specific survival.

To evaluate the importance of this pathway in the context of human PDAC and highlight the role in the formation of TME we scored our adenosine-related gene expression set against the PDAC subtypes published by Bailey.^44^ Of our 712 genes with a 50% decrease, 561 had a human ortholog and of these the z-scores of 517 genes were summed in each of the 97 patients with RNAseq data in Bailey *et al* to obtain a score. We were able to show that the adenosine-related gene expression is mostly expressed in the aggressive squamous subtype (figure 7A), that has been associated with a poorer prognosis. The score remained significantly higher even when comparing the squamous subtypes with the grouped non-squamous (figure 7B).

In order to create a PDAC-specific adenosine signature and to evaluate its performance in PDAC-specific outcomes, we created a signature using the 561 human orthologous genes with a ≥50% decrease in expression following Adoi treatment. Of these, 52 genes were selected for the signature (figure 7C), that according to our RNAseq data set were clearly associated with the pathway areas dependent on adenosine (hypoxia response, immunity, tumor stroma), were associated positively or negatively with PDAC prognosis, and were significantly co-expressed with CD73 and/or Adora2a in the PDAC genome data sets (TCGA or Bailey). Of the 176 and 170 patients with available PFS and DSS outcome, respectively, we found that the presence of a high adenosine signature is associated with higher probability of PDAC progression (median progression-free suvival, mPFS high Ado 13.05 vs low Ado 18.25, p=0.02; figure 7D) and a poorer PDAC-specific survival (median disease-specific survival, mDSS high Ado 19.66 vs low Ado *NR*, p=0.01; figure 7E), suggesting again that the presence of a functional adenosine pathway has a detrimental role in human PDAC. The signature was validated using the PDAC QCMG Bailey’s data set (49 genes were assessed, see figure legend) confirming a shorter overall survival in patients with high adenosine signature tumors (figure 7F p=0.003). It is worthy to consider that patients in the TCGA data set are predominantly non-metastatic, and the presence of the adenosine signature seems to become relevant for PDAC associated death 20 months after diagnosis. The presence of a high adenosine signature appears to be associated with shorter DFS, (median disease-free survival, mDFS high Ado 23.5 months vs low Ado 49.7 months, p=0.12; online supplemental figure 8M).

## Discussion

Overall our data highlight that the generation of the eAdo is instrumental for the innate immune system in shaping a protumorigenic, immune suppressive microenvironment in PDAC, in the context of a hypoxic milieu. We can speculate that this unfavorable environment may create the condition for a more aggressive PDAC phenotype which would then translate into the ability to escape the immune system, be resistant to cytotoxic treatment and metastasize readily.

PDAC is projected to become the second highest cause of cancer-related death in the USA within 10 years,^56^ and represents one of the major unmet needs of cancer treatment. Despite extensive efforts by laboratory and clinical scientists in the last 50 years, only 1% of patients diagnosed with PDAC today will survive for 10 years (https://www.cancerresearchuk.org/health-professional/cancer-statistics-for-the-uk).^1^ The response rate following standard treatments is poor, usually short-lasting, and associated with significant treatment-related toxicity.^2^ In the past few years, immunotherapy has provided new hope in the treatment of several types of cancer, and has dramatically changed the life expectancy of many patients with metastatic disease.^57^ However, this has not been true for patients with PDAC which is associated with a very low response rate to immunotherapy, usually confined to MSI-H/dMMR tumors, found only rarely in this disease.^3^

The role of the innate immune system in the generation of an immune suppressive/protumorigenic microenvironment in PDAC is well known. The presence of marked infiltration of macrophages has been identified as an independent predictive factor of the aggressiveness and prognosis of PDAC, in patients.^26 27^ Only recently, three phase I clinical trials in patients with PDAC have shown that targeting the innate immune system can have an impact in patients with PDAC. A phase I trial published on Lancet Oncology, showed that a combination of anti PD-1 and CD40 agonist, added to gemcitabine and nab-paclitaxel, led to 60% of response rate, with some durable responses, although the clinical benefit of adding a CD40 agonist to an anti PD-1 plus chemotherapy as first-line in patients with metastatic PDAC, was not confirmed in a phase 2 randomized trial.^58 59^ In addition, the inhibition of the CXCL12/CXCR4 axis has been demonstrated to modify the immunosuppressive TME of patients with PDAC and colorectal cancer.^60 61^

The eAdo pathway has also been shown to influence the TME fostering the immune suppression provided by some innate immune subpopulations (as myeloid and NKs) and inhibiting the function of the adaptive immune system, in particular, T cells.^7 9 52^ By activating its receptors, eAdo is able to increase the intracellular concentration of cyclic adenosine monophosphate (cAMP) which leads to the induction of an M2 phenotype of macrophages and block the secretion of interleukin (IL)-1β increasing the release of CXCL1, IL-6, IL-10 and IL-8 among others from myeloid population which are known to orchestrate immune exclusion.^11 62^ eAdo also favors the formation and maintenance of Treg cells,^11^ which are known to favor cancer progression and IOT resistance .

A recent publication, shows that genomic targeting on mouse PDAC cells of CD73 leads to a reduced in vivo tumor formation and change in the circulating and infiltrating immune system.^37^

However, to date, little was known about the expression of the adenosine pathway in the context of the innate and adaptive immune system in PDAC, how the eAdo is generated and what are the targets of adenosine also in regard to its spatial distribution and formation of adenosine.

Our results show that the mechanism of generation of eAdo in pancreatic cancer TME is finely orchestrated by tumor infiltrating myeloid cells and tumor cells, due to the expression of high level of CD39 in infiltrating myeloid cells and CD73 on both cell types. A recent paper demonstrated the presence of CD73 on MDSCs and macrophages.^36^ We identify that more broadly, ~90% of tumor-infiltrating myeloid cells in s.c. KPCY-derived tumors express CD73. We also validated this finding using an autochthonous tumor model as the KPC mouse. We have also shown that the pathway can be overexpressed in T cells infiltrating the tumors, regardless of their activation status (figures 1 and 2 and online supplemental figure 2 and 4). The distribution of eAdo is spatially heterogeneous and a high level of eAdo correlates with the presence of a hypoxic environment and is favored by the presence of necrosis, where the myeloid population is enriched (figure 3). Necrosis is common in human PDAC and related to poor prognosis for all stages.^63^ The enrichment of a CD39^+^ CD73^+^ double population, potentially able to independently produce adenosine, does not seem to correlate with IOT-resistant or responsive tumor models, but there is a difference when the target of adenosine (Adora2a receptor), is considered. Adora2a on myeloid populations, in particular in protumorigenic M2 macrophages but also in antigen presenting cells, is differentially expressed in regard of IOT response phenotype, with the resistant tumors abundantly overexpressing the receptor in these populations (figure 3 and online supplemental figure 4 and 5). Future analysis of human PDAC fresh specimens are needed to confirm the mechanism we propose in the context of human disease. The bulk RNAseq analysis of tumor treated with adenosine inhibition revealed indeed a broader role for adenosine in PDAC TME (figure 5). Genes related to immunosuppression and innate immunity recruitment (*Cd274*, *Csf2*, *Cxcl2*, *Ccl3*, *Ccl4*, *Ccl12*, *Il1a*, *Osm*, *Il6*), angiogenesis (*Vegfa*, *Vegfd*, *Adm2*, *Flt1*, *Pgf*, *Egln1*) and cell-ECM interaction (*Adam19*, *Adamts14*, *Adamts4*, *Adamts5*, *Col5a3*, *Col6a*, *Itga2*, *Itga7*, *Mmp3*, *Mmp9*, *Mmp12*) indicated the targeting of population of cells responsible for the acquisition of a protumorigenic, pro-metastatic, pro-fibrotic and immune resistant phenotype. Further, the downregulation of genes associated with hypoxia (*Hif1a*, *Hilpda*, *Nos2*, *Hk1*, *Hk2*, *Egln3*) following treatment shows not only that the adenosine pathway is induced during hypoxia (eg, CD73 and Adora2b), but that the hypoxia response is also dependent on the presence of the adenosine pathway in what we can speculate is a positive feedback loop. The inhibition of adenosine led to a reduced infiltration of M2 macrophages further from the hypoxic regions where adenosine is most abundant, suggesting that the effect of adenosine is stimulating the secretion of factors that are recruiting monocytes into the TME, replenishing macrophage infiltration (figure 6 and online supplemental figure 8). Notably, targeting the pathway can reduce tumor growth in an IOT-resistant model, improving the response to cytotoxic and immunotherapy combinations (figure 4 and online supplemental figure 7) even in historically therapy-resistant models such as the KPC mice.

Targeting adenosine would represent an alternative strategy to reduce the infiltration of protumorigenic macrophages in PDAC lesions. One approach has been the administration of a CSF1R inhibitor,^35^ which has shown promising preclinical data that have not been translated in humans so far. A recent publication shows that in CRC mouse models, the use of anti CSF1R treatment spares a subpopulation of macrophages characterized by the expression of *Cd274* (PD-L1), *Vegfa*, *Hilpda*, *Bhlhe40*, *Mmp12*, *Cebpb*, *Hmox1* among others.^31^ Given that all of these genes are among the immune suppressive and vasculogenic molecules that seem to be strongly downregulated by adenosine inhibition and our data show a reduction of some subpopulation of PD-L1^+^ macrophages, we can speculate that adenosine inhibition could potentially target these populations of pro-angiogenic, immune suppressive macrophages.

Information provided by the PDAC specific adenosine signature, indicate that the adenosine pathway appears to play a role in progression and survival of human PDAC, due to the ability of the adenosine inhibition to profoundly reprogram the TME in PDAC models (figures 5 and 6 and online supplemental figure 8). Gathering more information on the role of this pathway in human cancers should be a priority, retrospectively evaluating and then prospectively stratifying the patients on the basis of histopathological/radiological features and the spatial distribution of adenosine.

In summary, we have shown for the first time that tumor-infiltrating myeloid immune cells contribute to the generation of eAdo in the context of PDAC, correlating with the presence of hypoxia. Macrophages in particular, express high levels of Adora2a receptor in PDAC models and targeting the adenosine/myeloid axis remodels the TME. Data from IMC, flow cytometry and RNAseq suggest that the adenosine pathway is fundamental to the formation of a protumorigenic, immunosuppressive TME, and its expression is associated with an aggressive phenotype and poor survival in human PDAC. The remodeling of the TME caused by the inhibition of the adenosine pathway, translates into a delay of PDAC tumor growth. Finally, targeting eAdo should be considered as an adjunct to improve the efficacy of cytotoxic and IOT in future PDAC-specific clinical trials.

## Data Availability

Data are available in a public, open access repository. Data are available upon reasonable request. All data relevant to the study are included in the article or uploaded as supplementary information. The data generated in this study are available within the article and its supplementary data files or from the corresponding authors upon reasonable request. Code for differential expression analysis and visualization of RNAseq data is available via Github https://github.com/ka-lw/AdenoPDAC.
